# Incidence of side effects of antituberculosis drugs and their related factors in northern Iran: a retrospective cohort study

**DOI:** 10.3205/dgkh000482

**Published:** 2024-05-17

**Authors:** Motahareh Kheradmand, Mahdi Afshari, Mohsen Aarabi, Siavosh Abedi, Mohammadreza Parsaee, Asghar Nezammahalleh, Mahmood Moosazadeh

**Affiliations:** 1 Health Sciences Research Center, Addiction Institute, Mazandaran University of Medical Sciences, Sari, Iran; 2 Pediatric Gastroenterology and Hepatology Research Center, Zabol University of Medical Sciences, Zabol, Iran; 3 Department of Family Medicine, School of Medicine, Mazandaran University of Medical Sciences, Sari, Iran; 4 Department of Internal Medicine, Faculty of Medicine, Mazandaran University of Medical Sciences, Sari, Iran; 5 Health deputy, Mazandaran University of Medical Sciences, Sari, Iran; 6 Gastrointestinal Cancer Research Center, Non-communicable Diseases Institute, Mazandaran University of Medical Sciences, Sari, Iran

**Keywords:** antituberculosis drugs, adverse drug reaction, risk factor

## Abstract

**Background:**

Antituberculosis drugs may cause mild, moderate or severe adverse drug reactions (ADR) leading to poor compliance. Description of the pattern of ADR and their related factors can help tuberculosis (TB) control program as part of the WHO programs. This study aims to investigate the incidence of ADR and associated factors among TB patients in northern Iran.

**Methods:**

This is a retrospective cohort study. The required information, including year of diagnosis, age, gender, residence area, nationality, HIV co-morbidity, history of anti TB treatment and ADR, was obtained from the Deputy of Health, Mazandaran University of Medical Sciences, Iran. All data were analyzed using SPSS version 21 software.

**Results:**

Out of 3903 TB patients, 136 (3.5%) experienced major ADR. The incidence of ADR for men and women as well as for those with and without previous treatment history were 3.9% vs. 3.3% and 5.3% vs. 3.4%, respectively (p>0.05). Multiple logistic regression models showed a higher chance of ADR among those aged over 59 compared with those aged under 29 (OR=2.63, 95% confidence interval: 1.54–4.49).

**Conclusions:**

Age over 59 can be considered a risk factor for ADR with anti-TB drug administration.

## Zusammenfassung

**Zielsetzung:**

Antituberkulostatika (AT) können leichte, mittelschwere oder schwere unerwünschte Arzneimittelwirkungen (UAW) verursachen, die zu einer schlechten Compliance führen. Die Beschreibung des Musters von UAW und der damit zusammenhängenden Faktoren kann dem Tuberkulose(TB)-Kontrollprogramm helfen.

Ziel der Studie ist es, die Häufigkeit von UAW und die damit zusammenhängenden Faktoren bei TB-Patienten im Norden des Irans zu untersuchen.

**Methode:**

Es handelt es sich um eine retrospektive Kohortenstudie. Die erforderlichen Informationen einschließlich Diagnosejahr, Alter, Geschlecht, Wohnort, Staatsangehörigkeit, HIV-Komorbidität, Vorgeschichte der TB-Behandlung und UAW, wurden vom stellvertretenden Gesundheitsamt der Mazandaran Universität für Medical Sciences, Iran, bereitgestellt und mit der Software SPSS Version 21 ausgewertet.

**Ergebnisse:**

Von 3903 TB-Patienten traten bei 136 (3,5%) schwerwiegende UAW auf. Die Häufigkeit von UAW bei Männern bzw. Frauen betrug bei vorheriger Behandlung 3,9% bzw. 3,3%, ohne vorherige Behandlung 5,3% bzw. 3,4% (p>0,05). Multiple logistische Regressionsmodelle ergaben, dass die Wahrscheinlichkeit einer UAW bei über 59-Jährigen höher war als bei unter 29-Jährigen (OR=2,63, 95 % Konfidenzintervall: 1,54–4,49).

**Schlussfolgerung:**

Ein Alter über 59 Jahren kann als Risikofaktor für UAW gegen Antituberkulostatika angesehen werden.

## Background

Despite the reduction in tuberculosis (TB) incidence, its morbidity and mortality is still one of the main global public-health concerns. The annual incidence and the mortality of TB has been estimated to be 9.6 million and 1.5 million, respectively [1], [2]. The first line anti-TB drugs (Rifampin, Isoniazid, Pyrazinamide) have an efficacy exceeding 95% [3]. 

Anti-TB drugs may cause some adverse drug reactions (ADR), varying from mild to severe forms. Just 2%–8% of TB patients may experience severe ADR, such as exanthema, vertigo, psychosis and hepatotoxicity, leading to a termination of or change in treatment regimen. Conversely, mild to moderate ADR, including gastroenteric problems, nausea/vomiting, arteritis, peripheral neuropathy, drug allergy, rash/itching, headache and behavioral problems (insomnia, anxiety, hypolibido), do not require emergent change in treatment regimen. Such complications may challenge the TB control program [4], [5], [6], [7], [8]. Hepatotoxicity, one of the most severe drug reactions, occurs in the first month of treatment and can be fatal if diagnosed late [2]. The ADR incidence is also affected by dosage and time of drug prescription. Age, nutritional status, co-morbidities such as liver or renal dysfunction as well as HIV infection and alcoholism are other determinants for TB ADR [6].

In addition to high burdens associated with ADR for patients and communities, diagnosis and treatment of such complications cause high economic costs, including hospitalization, provision of drugs and food supplements, and a negative impact on the work force [4]. Describing the pattern of ADR onset along with investigating the factors associated with such adverse reactions can help policymakers to control and manage the relevant costs [9]. In this study, we aimed to determine the incidence of ADR and relevant risk factors among TB patients in Mazandaran University of Medical Sciences. 

## Methods

The retrospective cohort study was conducted among 3,903 patients with TB treated with anti-TB drugs from 2005 to 2017. All of them were recruited by census method. Inclusion criteria were: all TB patients who registered and were followed-up from treatment initiation until the end of the second month of treatment. Exclusion criteria include:

1. Subjects who were diagnosed and registered as TB patients but later on TB diagnosis was ruled out.
2. Patients who were diagnosed in another center and referred to the TB registry system of Mazandaran University of Medical Sciences, but their history of adverse-reaction experience was not available.

The required information was provided from the TB registry system by the Health Deputy of Mazandaran University of Medical Sciences, Sari, Iran, in excel format. This information included year of diagnosis, age, gender, area of residence, nationality, HIV co-morbidity, history of anti TB treatment and ADR. The following adverse reactions were assessed: peripheral neuropathy (burning of the extremities), nausea, vomiting, abdominal pain, edema, mucosal ulcers, shock, hearing loss or deafness, vertigo, nystagmus, icterus, visual impairment, acute liver failure, thrombocytopenia, acute renal failure, feverless skin rashes and skin rashes with fever. 

Adverse reactions were evaluated and approved by a general practitioner who was in charge of the treatment of TB patients. According to the WHO Tuberculosis Control Program, we defined the presence of ADR as at least one of the above-mentioned side effects in patients receiving tuberculosis treatment. In the case of any missing data, the research team was referred to the peripheral city districts and collected the required data from the patients and their files. 

The collected data were transferred to SPSS version 21 after refinement. The incidence of ADR and different types of the reactions were described by percent frequency. Univariate and multivariate logistic regression models (adjusting for potential confounders) were used to test for the association between ADR onset and different factors. A p-value less 0.05 was considered statistically significant. 

This study was approved by the ethics committee of Mazandaran University of Medical Sciences (IR.MAZUMS.REC.1398.36). Written informed consent was obtained from all participants. 

## Results

During the study period, 4,033 TB patients were registered, 130 of whom were excluded (86 patients due to wrong diagnosis and 44 cases were transferred from other regions). Of the 3,903 remaining patients, 136 (3.5%) had experienced adverse drug reactions during treatment. The type of drug reaction was identified and reported in 107 patients. Of these, 92 had one ADR, 13 patients reported two types, and 2 patients had three types of ADR. Renal adverse reaction, vertigo, vomiting, icter, feverless skin rashes and also skin rashes with fever were observed in 9, 4, 23, 77, 6, and 5 patients, respectively. 

Univariate analyses showed that ADR frequency was higher among women than men (3.9% vs. 3.3% respectively), urban residents than rural residents (3.7% vs. 3.2% respectively), patients with previous treatment history vs. those without (5.3% vs. 3.4% respectively), HIV-positive TB patients vs. HIV-negative TB patients (5.9% vs. 4.3% respectively). None of these associations were statistically significant (p>0.05). The frequency of ADR among patients aged >59 was significantly higher than that among patients aged <30 (5% vs. 2% respectively, p=0.001). Using a multivariate logistic regression model and controlling for possible confounders, only age >59 significantly increased the odds of developing ADR (OR=2.633, 95% CI: 1.54–4.49) (Table 1 (Tab. 1)). It should be noted that there were no differences between the effect size of multivariate logistic regression and the univariate model. Moreover, there was no potential confounder.

## Discussion

The results showed that 3.5% of TB patients in northern Iran experienced adverse drug reactions during anti-TB treatment. Although these reactions were higher among women and re-treatment cases, the associations were not statistically significant. Multivariate logistic regression models showed that out of the investigated factors, only age over 59 compared with age under 30 was significantly associated with ADR. 

The rate of ADR in the present study was lower than those reported in studies performed in India [10], China [1], Brazil [11] and the Markazi province in Iran [12]. It should be noted that demographic characteristics and methods of data registry were different in these other study regions. In addition, different surveillance systems, ethnicities, study designs and various definitions of ADR were other factors responsible for these heterogeneities [13]. 

Some of the previous studies reported higher ADR incidences among men [10], while others reported that women were more affected than men [1], [11], [12]. Although the present study reported that ADR was more common among women, the difference was non-significant. Higher adverse reactions among women might be due to hormonal fluctuations during different periods of their lives. Moreover, interactions between oral contraceptives and anti-TB drugs might be another reason for such an association [14]. 

In the current study, icterus was the most common adverse reaction, while digestive complications and hyperuricemia were reported in some other studies [1], [10], [11]. Hepatic and digestive problems were reported in the study conducted among Chinese patients [15]. 

Our study revealed that age is related to adverse drug reactions during anti-TB treatment, which is in keeping with the results of several other studies [1], [10], [12]. However, results of a meta-analysis did not report this factor as a risk factor for ADR [16].

### Limitations

One of the limitations of the current study is using registry data of a medical university, which is not collected according to research purposes and is prone to some defects and biases. Therefore, it was not possible to assess the role of some factors such as weight and diabetes co-morbidity in developing ADR. Further studies are recommended to prospectively investigate the association between all relevant factors with ADR onset. The high possibility of underreporting adverse reactions was another limitation of the present study.

## Conclusions

This study shows that age >59 can be considered as a risk factor for ADR to anti-TB drugs.

## Notes

### Competing interests

The authors declare that they have no competing interests.

### Acknowledgments 

The authors thank Mazandaran University of Medical Sciences for the kind financial support and also the Health Deputy of this University for providing the necessary data.

### Funding

This study was carried out with the financial support of Mazandaran University of Medical Sciences (grant No. 1398.36).

### Authors’ ORCIDs

- Kheradmand M: 0000-0002-4843-302X
- Afshari M: 0000-0002-3159-8741
- Aarabi M: 0000-0003-3811-8306
- Abedi S: 0000-0003-4453-3661
- Moosazadeh M: 0000-0002-5452-514X

## Figures and Tables

**Table 1:**
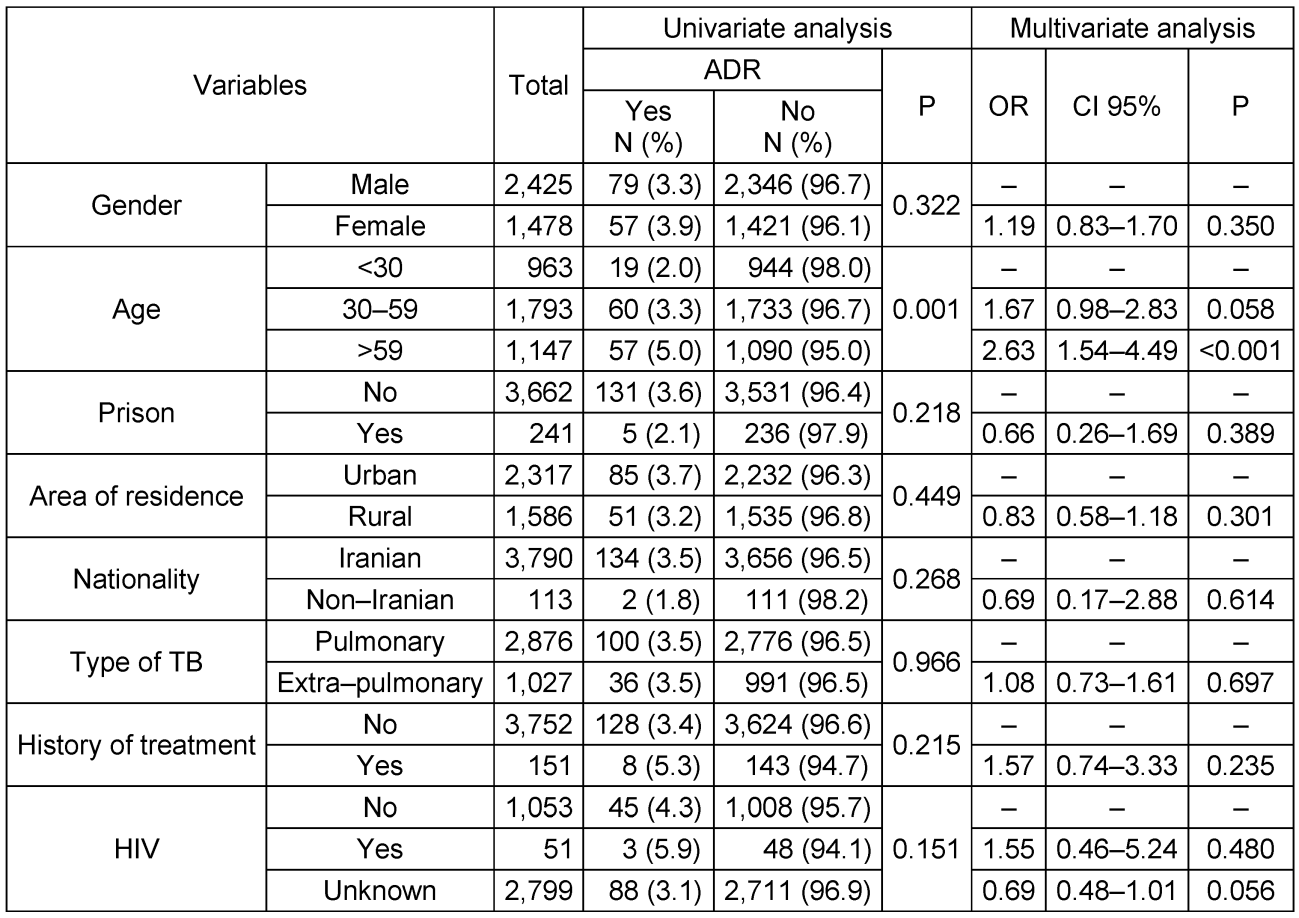
Factors related to ADR based on univariate and multivariate analysis
